# Trace element & vitamin content of Ankaferd hemostat and hepatoprotective effect

**DOI:** 10.3906/sag-2109-30

**Published:** 2021-10-30

**Authors:** Begüm GÜNEŞ, N. Yasemin ARDIÇOĞLU AKIŞIN, Nejat AKAR

**Affiliations:** 1 Faculty of Medicine, TOBB University of Economics and Technology, Ankara, Turkey; 2 Department of Biochemistry, Faculty of Medicine, TOBB University of Economics and Technology, Ankara, Turkey; 3 Department of Pediatrics, Faculty of Medicine, TOBB University of Economics and Technology, Ankara, Turkey

Dear Editor,

We read with great interest the article entitled “The effect of Ankaferd blood stopper on liver damage in experimental obstructive jaundice” which appeared recently in the Journal [1].

It has been previously suggested that Ankaferd hemostat (ABS; Ankaferd Blood Stopper, İstanbul, Turkey) was not only a hemostatic agent but a pleiotropic agent with antioxidative, antiinflammatory, antineoplastic, antithrombotic, antiinfective, antifungal, and wound healing effects [1–6]. Antioxidative, antiinflammatory, and, therefore, hepatoprotective effects of ABS may be associated with its trace elements and vitamins. In this paper, we present the trace elements and vitamins in ABS that might carry antioxidant/hepatoprotective properties (Table) [7–10].

Magnesium and calcium values were measured spectrophotometrically by Elecsys e401 (Roche Diagnostics Co., Mannheim, Germany). Vitamin D and vitamin B12 values were determined by electrochemiluminescence immunoassay using hormone analyzer Elecsys e501 (Roche Diagnostics Co., Mannheim, Germany). Vitamin B9, vitamin A, and vitamin E were analyzed by high performance liquid chromatography (HPLC) (Agilent Technologies, Inc. Santa Clara, USA).

In previous studies on trace element and vitamin content of ABS, Akar et al. investigated the Fe (III), Cu (II), Zn (II), and Ag (I) ions in ABS. Concentrations were 2163 ± 7, 2.56, 9.2, and 45.0 ppm, respectively and were suggestive of a link between high iron levels and the hemostatic action of ABS. Also, their study indicated the absence of Pb (II), Ni (II), Cr (IV), Co (II), and Cd (II) ions in ABS [3]. Koluman et al. isolated various antioxidant molecules, including members of the Vitamin E family [4]. Ardıçoğlu Akışın et al. analyzed the Zinc concentration to be 300 μg/dL and presented the link between high zinc levels and the wound healing action of ABS [5].

Due to the possible antioxidative and hepatoprotective effects of the trace elements and vitamins like magnesium, calcium, vitamin D, vitamin B12, vitamin B9, vitamin A, and vitamin E [7–10]; further detection of the trace elements and vitamins of ABS can give insight into understanding the mechanisms of the pleiotropic effects mentioned above (antioxidative, anti-inflammatory, etc.), along with forming the whole composition of the agent itself.

## Figures and Tables

**Table:** Trace element and vitamin content of Ankaferd hemostat.

Trace element / Vitamin	Concentration
Magnesium (Mg)	5.11 mg/dL
Calcium (Ca)	1.0 mg/dL
Vitamin D (25-Hydroxycholecalciferol)	3.0 ng/mL
Vitamin B12 (Cobalamin)	434 pg/mL
Vitamin B9 (Folate)	> 20 ng/mL
Vitamin A (Retinol)	< 5 μg/dL
Vitamin E (Alpha Tocopherol)	< 0.1 mg/dL
